# Ordering of computed tomography scans for head and cervical spine: a qualitative study exploring influences on doctors’ decision-making

**DOI:** 10.1186/s12913-022-08156-2

**Published:** 2022-06-18

**Authors:** H. Laetitia Hattingh, Zoe Alexandra Michaleff, Peter Fawzy, Leanne Du, Karlene Willcocks, K. Meng Tan, Gerben Keijzers

**Affiliations:** 1 Diagnostic and Sub-Specialty Services, Gold Coast Health, Southport, Gold Coast, QLD 4215 Australia; 2 School of Pharmacy and Medical Sciences, Griffith University, Southport, Gold Coast, QLD 4222 Australia; 3 Institute for Evidence-Based Healthcare, Bond University, Gold Coast, QLD 4229 Australia; 4 Neurosurgery Department, Gold Coast Health, Southport, Gold Coast, QLD 4215 Australia; 5 School of Medicine and Health Sciences, Bond University, Gold Coast, QLD 4226 Australia; 6 Medical Imaging, Gold Coast Health, Southport, Gold Coast, QLD 4215 Australia; 7 Department of Emergency Medicine, Gold Coast Health, Southport, Gold Coast, QLD 4215 Australia; 8 Faculty of Health Sciences and Medicine, Bond University, Gold Coast, QLD 4226 Australia; 9 School of Medicine, Griffith University, Southport, Gold Coast, QLD 4222 Australia

**Keywords:** Computed tomography scan, Emergency doctors, Head and cervical spine trauma, Low value care, Choosing wisely, Decision support

## Abstract

**Background:**

Ordering of computed tomography (CT) scans needs to consideration of diagnostic utility as well as resource utilisation and radiation exposure. Several factors influence ordering decisions, including evidence-based clinical decision support tools to rule out serious disease. The aim of this qualitative study was to explore factors influencing Emergency Department (ED) doctors’ decisions to order CT of the head or cervical spine.

**Methods:**

In-depth semi-structured interviews were conducted with purposively selected ED doctors from two affiliated public hospitals. An interview tool with 10 questions, including three hypothetical scenarios, was developed and validated to guide discussions. Interviews were audio recorded, transcribed verbatim, and compared with field notes. Transcribed data were imported into NVivo Release 1.3 to facilitate coding and thematic analysis.

**Results:**

In total 21 doctors participated in semi-structured interviews between February and December 2020; mean interview duration was 35 min. Data saturation was reached. Participants ranged from first-year interns to experienced consultants. Five overarching emerging themes were: 1) health system and local context, 2) work structure and support, 3) professional practices and responsibility, 4) reliable patient information, and 5) holistic patient-centred care. Mapping of themes and sub-themes against a behaviour change model provided a basis for future interventions.

**Conclusions:**

CT ordering is complex and multifaceted. Multiple factors are considered by ED doctors during decisions to order CT scans for head or c-spine injuries. Increased education on the use of clinical decision support tools and an overall strategy to improve awareness of low-value care is needed. Strategies to reduce low-yield CT ordering will need to be sustainable, sophisticated and supportive to achieve lasting change.

**Supplementary information:**

The online version contains supplementary material available at 10.1186/s12913-022-08156-2.

## Background

Emergency Department (ED) doctors need sound clinical decision-making skills to evaluate patients’ status and guide their management. Many patients require additional laboratory or imaging tests to refine the working diagnosis. Although computed tomography (CT) scans have good diagnostic accuracy [1] concerns have been raised about the resource utilisation and health care costs associated with these scans [2–5] and the potential harms of radiation exposure, especially in young patients [6, 7]. There may also be adverse consequences of overdiagnosis, i.e., diagnosis and subsequent treatment of clinically insignificant disease or issues not related to the health encounter [8, 9]. Furthermore, when service capacity is constrained, providing low-value care (interventions where evidence shows they provide no patient-centred benefit) to one patient can delay provision of care to another patient, resulting in indirect harm [10].

Several factors influence clinicians’ decisions when ordering tests. These include clinician factors (fear of missing a diagnosis; others’ clinical expectations), environmental factors (competing interests and distractions) and patient factors (expectations, knowledge and health literacy) [11, 12]. Evidence suggests that both doctors and patients tend to overestimate the benefits and underestimate the harm of medical interventions [13, 14].

Various clinical decision support tools, also referred to as clinical decision rules or aids, are available to guide ED doctors in their decision-making [15, 16]. The Canadian CT Head Rule (CCHR), [17–19] New Orleans Criteria [20] and National Emergency X-Radiography Utilization Study (NEXUS) II [21] are validated clinical decision tools which can be used to guide imaging decision making in patients with a head injury. The Canadian Cervical-spine (C-spine) Rule (CCR) [22] and the NEXUS criteria [23] are validated clinical decision support tools [24] to guide imaging decisions in patients with C-spine injuries. The use of validated decision support tools is recommended by the Royal Australian and New Zealand College of Radiologists and Australasian College for Emergency Medicine to reduce unnecessary scans [25].

Studies that involved auditing of CT scan orders against criteria in decision support tools showed varying degrees of compliance. Retrospective [26] and prospective studies [27] showed between 16 and 29% of C-spine CTs were unnecessary according to NEXUS or CCR criteria. Similarly, an audit of CT head scans found 27% were likely unnecessary [28] whilst another study in younger patients (18–45 years) found up to 72% of orders were unnecessary [29].

In the context ever-increasing demand for medical imaging, we sought to understand the influences on ED doctors’ CT ordering decisions to inform remedial strategies.

## Methods

### Study design

A qualitative approach was followed consisting of semi-structured interviews with ED doctors. The interviews were conducted between February and December 2020. We used the COREQ checklist and qualitative research criteria in the development, analysis, and reporting of the study [30, 31]. This study was approved by the Gold Coast Hospital and Health Service and the Bond University Human Research Ethics Committees (LNR/2019/QGC/58433).

The primary outcome relates to thematic inductive analysis to identify key underlying factors influencing the ordering of CT scans by ED doctors and their decision-making processes followed by a mapping of sub-themes to the COM-B, a behaviour change model. Facilitators and barriers regarding the use of clinical decision support tools are reported separately.

### Setting

ED doctors working in one of two public hospitals within a Hospital and Health Service in Queensland, Australia*.* Acute and unscheduled care delivered in Public EDs in this health service and in Australia is covered by the national insurance scheme – Medicare. There is no financial (dis)incentive for clinicians to order scans.

### Participants

Doctors working in either ED were purposively approached to represent varying levels of experience, roles (e.g., educator, researcher) and age groups. Purposive selection of potential participants allowed for maximum variation in the sampling whilst enabling in-depth inquiry into the topic of interest [32]. This approach was followed to obtain information rich data and improve the reliability and credibility of the research findings [33]. Recruitment involved using the researchers' networks and subsequent snowballing [34].

Clinicians were invited by email between January and November 2020. Respondents were provided a study Participant Information and Consent Form and private one-on-one interviews were conducted in person, via MS Teams or by telephone. Participant demographic information was collected before interviews commenced. The interviews were conducted by the same interviewer (LH) who is experienced in qualitative interviews to ensure consistency. Interviews were audio-recorded with supplemented notes handwritten by the interviewer, transcribed verbatim by an independent party, de-identified, and quality checked. Interview participants had the opportunity to check their transcripts.

Taking into consideration studies exploring the number of interviews required to reach saturation, [35] the purpose of the research [34] and the research team’s experiences it was estimated that between 15 to 25 interviews had to be conducted, depending on when data saturation was reached.

### Interview tool

A tool (see supplementary material) to guide semi-structured interviews was developed considering the literature on qualitative interview tools, [33, 36] behaviour change components [37] and team members’ expertise. The interview tool utilised both pre-determined open questions and the opportunity for the interviewer to explore particular themes or responses further and adapt questions as conversations progress [38]. It consisted of 10 questions with prompts to explore participants’ ordering practices and incorporated three hypothetical patient scenarios to facilitate discussions on decision-making processes. The interview tool was iteratively developed and tested for face and content validity through feedback from three researchers with expertise in qualitative interviews and two clinicians with ED experience.

### Coding framework

The development of the coding framework involved a systematic process of allocating participant quotes to codes. Codes were then grouped to form categories, which subsequently formed sub-themes, exploring aspects that influence ordering practices and decision-making processes. The subthemes were then rearranged to represent emerging topics which were used to develop an analytical framework. Two researchers (LH and ZM) did the initial coding and early in the coding process the framework was discussed with team members GK and LD (senior ED doctor/researcher and senior radiologist) with agreement on the coding framework. All research team members reviewed the analytical framework and considered it finalised when no new ‘themes’ or ‘ideas’ emerged in addition to the existing codes and categories and all discrepancies had been resolved. Data saturation was the point at which the gathering of more data revealed no new insights, and no new codes were identified.

Subsequent to the thematic analysis, the themes and sub-themes were mapped against the Behaviour Change Wheel [37]. The Wheel has the COM-B model of behaviour at the centre, representing three essential components namely Capability, Opportunity and Motivation (COM), to provide a framework for understanding Behaviour (B). This process was incorporated to provide a basis for future interventions.

### Data analysis

An iterative process was followed with data collection and analysis taking place simultaneously. This allowed the interviewer to adopt the interview guide throughout the study to explore emerging issues. Transcripts were de-identified and imported into NVivo Release 1.3 (QSR International Pty Ltd) for organisation of the data and data analysis. Two researchers (LH and ZAM), both clinical researchers with experience in qualitative research, analysed the data independently following an inductive approach to facilitate thematic analysis [39]. During the data analysis phase, the two researchers had regular meetings to discuss disagreements and reach consensus with regular communication with all team members. Transcripts were read repeatedly by the researchers to gain a deep understanding of the topics discussed before initial ideas were coded as 'nodes' under the coding framework.

## Results

Researchers were confident that data saturation had been achieved after 21 interviews (19 face-to-face, one each via MS Teams and telephone). Mean interview duration was 35 min (range, 23–49). Participants ranged from first year interns to experienced consultants with up to 14 years experience as an ED doctor. Seventeen worked full-time in clinical roles with the remaining four in joint clinical, research, education and digital development roles. Table 1 summarises participants’ demographic data.

**Table 1:** Participant demographic details

Position	Gender		Where completed medical education	
	**Male**	**Female**	**Australia**	**Overseas**
Consultant: *n* = 6	5	1	2	4
Registrar/Resident: *n* = 10	8	2	4	6
Intern: *n* = 5	2	3	3	2

Five overarching themes emerged from the data: 1) health system and local context, 2) work structure and support, 3) professional practices and responsibility, 4) reliable patient information, and 5) holistic patient-centred care. Example quotations are used throughout to contextualise the findings with the following identifiers: consultant (C), registrar or resident (R) and intern (I).

### Health system and local context

External health system factors, both national and local, influenced CT ordering (Table 2):

- *Australian practices*
- *Resources*
- *Bed flow*
- *Costs*

**Table 2:** Health system and local context impacting on the ordering of CT scans

National Health System	
Australian practices	*… the Australian doctors that I worked with there certainly are more risk averse and I think that they're just so scared of whether it's being sued or whether it's what their peers will think or I don't know where the feeling comes from, but they certainly have a much lower threshold to image people, to do advanced imaging like CT of any part of the patient really, but specifically brain.* P6 R *The specialty of emergency medicine in Australia involves looking after patients more definitively and for a longer period of time than UK where it's more of a triage system. There's more of an emphasis in the UK of which destination the patient's going to in terms of home or surgeons or medicine or so on, whereas here we're involved in more long-term care of patients. … we have greater access to imaging in Australia … compared to the UK … But there was also a lot more oversight of CT cross sectional imaging, so all scans had to be approved by a radiology registrar so they couldn't be ordered by an emergency physician. Here non contrast scans for CT can be ordered by a consultant during the day or a registrar … overnight and scans with contrast need to be approved.* P13 R
**Local System**	
Resources	*I suppose access to resources, I mean, I think I may have touched on this already, but we are very lucky here on the [health service] that we’ve got excellent facilities and access to them 24/7. Sometimes it’s a little bit different in [smaller hospital] for example where you haven’t got CT on site*. P1 R *They're [CT scans] so easy to do and we have such easy access to them and I wonder if having a CT scanner in the ED makes it easy. That's great for when you have trauma and stuff, but I wonder does that also encourage misuse of it. … I don't want to work in a system where I have to fight for every scan, but I do think that there needs to be some policing that we're not just running these people through the scanner again and again without any benefit to the patient*. P6 R
Bed flow	*but I must admit, when I do make decisions on when to scan someone's—do a CT head if they've had a fall, in an older patient sometimes I do decide to scan more readily if it's going to aid a faster disposition for that patient rather than to need a four-hour period of observation.* P7 C *If we'd decided to scan that patient, they would have waited for the CT scan itself, it might be an hour; they would have waited for the result of the CT scan, that might be another hour. That adds two hours for them staying in the department, it's an area that then can't be used for other patients, so it does have cost implications, but even more than that it has implications on flow for the department and that is I think very important. Sometimes in certain patients it can be a helpful thing to get a scan done early because it helps facilitate their discharge but in other patients it holds them in the department and disrupts the flow.* P13 R
**Cost**	
Admission and lenghth of stay	*If the scan is going to enable an early disposition for the patient, I think it's going to be cheaper to do the scan than to have them clog up a bed for four hours and then require further follow-up potentially in the community as well in terms of GP follow-up.* P7 C
Braod sense	*I do think about cost and time, and it's not just the individual aspect of that particular patient. Because the Department as a whole, for example, you only have one CT scanner. Scanning one person that may not need it means that you're delaying a scan for someone else and that's all money and time as well. Every minute spent in the ED is also money, but yet again the scan could cost a few hundred dollars, but that could mean as well [to] save time for the patient to be in the ED which is also a lot of money. … So you just go by what you think it’s probably the most efficient way without a lot of information. … It's not just whether we can do something about it surgically, even the outside hospital aspect does cost money if the family's not getting closure, they've got a lot of stress in their life from it, they are not working and it has other impacts beyond, I feel.* P21 R

Participants who previously worked in the United Kingdom (UK) discussed differences between UK and Australian *health practices*, specifically commentingthat CT ordering scans by ED doctors is more frequent in Australia than in the UK. Another key difference was the requirement for radiologist approval:

*Compared to working in the UK; in the UK you’d go begging to a radiologist and most of the requests would be rejected, whereas here it’s no issue whatsoever getting a scan. In fact, for most CTs you don’t have to speak to anyone about getting a scan, you just request it and it goes through.* P15 C

From a local perspective, ordering of a CT scan was influenced by the availability of *resources*. Participants with experience working at the the smaller of the two hospitals, or in a rural and remote hospitals, highlighted how the availability of a CT scanner, radiologist and radiographer influenced the decision to order a CT scan and the timing of this decision:

*… it’s just because we’re in a huge trauma centre with excellent facilities that perhaps some scans I get here, maybe I wouldn’t get back home.… You have a lot more influences in your decision-making when you haven’t got the easy access to resources I suppose. I would always still call nursing homes and if it’s an appropriate time, call the next of kin, even if working in [bigger hospital]. I think in [smaller hospital] … you have to take in the fact that you can’t get a scan within 20 minutes like you can here if you’re concerned*. P1 R

A few participants explained practices of ordering scans for elderly patients from Residential Aged Care Facilities (RACF) to facilitate return to the RACF:

*However, depending on how settled she is in the Emergency Department, you may end up scanning her to get her back to the nursing home more quickly, in that the radiation from the CT scan is not going to be an issue for her but getting her out of this busy unfamiliar environment would be good given that patients like this in hospitals get injured. They fall, they freak out.* P5 R

More experienced doctors discussed the impact of *bed flow* and criteria to admit or discharge patients within four hours of presentation. There were varied opinions as to whether CT ordering would shorten or lengthen patient stay, although there was general agreement that length of stay and patient stress were important considerations.

Views about *cost* considerations were wide-ranging with some not mentioning cost at all, while others considered it alongside other costs of admission and length of stay. One participant considered cost in a more holistic and broader sense for the Department as well as the ongoing care of the patient and potential impact on the family.

### Support structures and resources

Support structures guiding decision-making incorporated local and online resources, with five sub-themes identified (Table 3):

- *Consult with senior ED doctors*
- *Consult with radiology staff*
- *Model consultants and more experienced doctors*
- *Ongoing training*
- *Clinical decision support tools*

**Table 3:** Sub-themes relating to support structures in place to guide decision-making

Consult with senior	*… if you’re unsure you will tend to have more in-depth conversation with them about, ‘look I’m really not sure’. Sometimes they’ll actually go and see the patient and decide and sometimes they’ll just have a longer chat with you about what you think*. P2 R *Generally, CT scans for head, or C-spine, we are recommended to often talk to the consultant before we order them, so we get a second opinion before we order them, because of the high risk of radiation. Yes, so we will make the initial decision, because we've seen the patient, we [worked] them up from point A to point B, but we always like to get a confirmation from the consultant*. P9 I
Consult with radiology staff (registrar/ radiologist/ radiographer)	*There's been many discussions with radiology registrars, especially on night shifts, around whether or not to do scans*. P5 R *I've been relying on them [radiology registrar] a lot more to help that decision-making because there are times when I would want to order something and they say ‘no, that's not appropriate’ or ‘that's not going to show you anything or a CT is not going to help in your management, it's not going to change your management’*. P11 I
Model consultants and more experienced doctors	*We see that amongst consultants, as well. Especially things like one of the criteria is if a patient has a painful distracting injury, probably one of the most equivocally applied criteria in NEXUS, some consultants will think of it as if a patient's distracted. Others will think of it as if the patient, either the patient has a distracting injury, regardless of whether they're distracted by it or not. That difference in opinion is very common amongst senior doctors. As junior doctors, we often listen, wonder why the consultant thinks so. Often, they will apply their experience, more so than the criteria*. P9 I
Ongoing training	*There is one big period in an emergency trainee’s life, particularly in Australia … is when you study for your Fellowship exams, it does change significantly the way you practice. Because up until that point you can be a very good clinician, you can be a very experienced clinician, but you only realise when you study for that exam, the knowledge that you’re missing. And that does inform, I think – there’s several things, not even that I’ve purposely changed, but having the background knowledge of a specialty training exam, a consultancy does make a difference in that respect*. P15 C
Clinical decision support tools	*But where I would use it is in those middling patients, where I’m not sure.* P15 C *it's a matter of incorporating both the experience and decision rules. At this moment, at this point in time, as a junior doctor, I trust the decision rules more than I trust my experience, because due to lack of my experience at this stage.* P9 I *The clinical decision-making tools do two things; I think they can confirm what you're thinking, so they can support your professional opinion, but they can also help as a checkpoint to make you rethink what you're doing as well.* P18 R

In addition to support from other clinicians and the health service, all participants used *clinical decision tools* to support their decision-making and clinical judgement, particularly in patients without clear-cut injuries. The stage at which the clinical decision support tools were referred to or integrated in the decision-making process differed amongst participants:

*I tend to keep both [clinical decision support tools] in mind when I'm assessing the patient.* P3 C

*I use the tools alongside my clinical judgment. I don’t use the tools to decide 100 per cent. The tools help me if I'm unsure, I don’t consult them every single time*. P6 R

*… and that sometimes there were more difficult decisions falling on my head, which has been challenging at times, and I guess it's just been a note to myself to, if I'm in doubt, check; check the guidelines*. P13 R

A few participants discussed the need for appropriate training in the use of clinical decision support tools e.g., when to use the various aids, how to apply the algorithms, understanding limitations, sensitivities and specificities:

*But the only concern I have, I guess, is that they're not 100 per cent sensitive and specific. They're all validated in different populations. You also must have people trained in how to use them and who is an appropriate population and patients to use them on*. P6 R

Of interest was that more experienced doctors automatically incorporated criteria from various clinical decision aids into their decision-making, like being on ‘autopilot’. They seemed to be less focused on individual steps but more on the patient’s holistic picture compared to more junior doctors:

*Then of course the use of clinical decision rules is something that we pretty much practise every single day. I think we don't even realise that we're doing that because most of those things just form part of the history and the exam that you do anyway*. P3 C

### Professional practices and responsibility

**Fig. 1:**
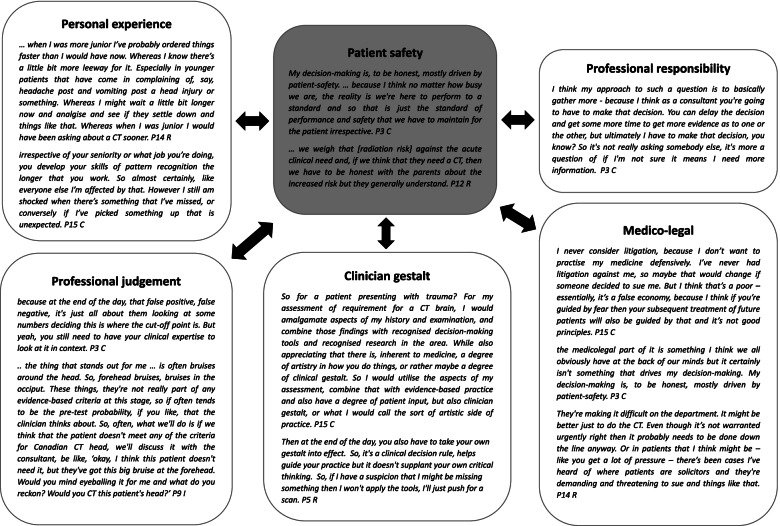
Sub-themes related to professional practices and responsibility that impact on ordering of CT scans

Doctors’ *professional responsibility* and requirement to meet professional standards were integrated throughout participants’ comments. More senior participants relied on *personal experiences* and *clinician gestalt* to guide CT neck and C-spine ordering decision-making processes, whilst more junior doctors relied more on clinical decision aids. A consistent thread was the need to apply *professional judgement* and accept professional responsibility for patients. Consultants considered *medico-legal issues* however did not feel pressured to practise defensive medicine. One participant commented on potential litigation pressures placed on clinicians by patients or family members that impact decisions to order scans in some situations (Fig. 1).

*Patient safety* emerged as a central sub-theme related to professional practices and responsibility. Radiation risk was discussed by most participants, especially in younger patients as well as older patients with multiple prior scans. There was widespread recognition that patients/parents must be informed of these risks. Most participants referred to considering a patient’s age and that they were specifically cautious in scanning younger patients due to lifetime exposure to radiation:

*Definitely we consider radiation risk in children. That’s the contrast I was attempting to highlight. A 90-year-old, the radiation risk is essentially non-existent, whereas a 12-year-old, you’ve always got to think about the lifetime, increased risk of cancer.* P12

Participants generally believed CT should not be ordered unless it would guide/change management, although this concept was applied quite broadly:

*So in a case with a patient who likely has an advance health directive, has severe dementia and has reduced cognitive function, one could argue, probably quite reasonably from an ethical perspective, that irrespective of the result of the scan it won’t change what we do for the patient. However, it may be of use for the nursing or the family to know that – the scan may be useful to say ‘look your mother or father doesn’t have a severe brain injury and they’re going to go back to the nursing home’. Or you might be able to say, ‘well there is a bleed here – we’re not going to do anything about it, but this may get worse over the next few days’. So then you’re introducing things like the family’s understanding of the case, being able to manage the patient in the nursing home rather than the patient being sent back to hospital two days later when she deteriorates. So even though you may not be directly intervening as a result of the scan, the prognostication can help with the management of the patient.* P15 C

However, the risk of incidental findings was recognised:

*inappropriate tests, a consequence aside from harm of the patient, is that you might [have] results that are uninterpretable, because there’s no context to them*. P15 C

### Reliable patient information

Participants identified the need to gather reliable information to determine a patient’s baseline level of risk considering known and unknown factors that would guide CT ordering:

*So, for me, any assessment always begins with history and examination and putting together pre-test probability for what investigations that I’ll be ordering to help answer a defined clinical question*. P16 C

Participants discussed a process of ‘information gathering’, ‘analysis’ and ‘evaluation’ guided by history taking, including collateral history, and physical examination that occurred against a prioritised list of differential diagnoses that was then used to estimate a patient’s baseline level of risk and determine their need for a CT scan. Although participants aimed to be objective in their decision-making, cognitive bias impacted decision making in certain situations:

*I wouldn't trust the history as well, given that the patient is still smelling of alcohol. Even though the patient may not be intoxicated in their behaviour, but they’re smelling of alcohol is something that many studies would classify as still being intoxicated and still makes the patient a bit, I guess, an invalid source of information*. P9 I

*… are they a reliable historian. So if the patient is actually not - for example if it's an advanced dementia patient it's very hard to make a clinical decision based on them because they can't tell you anything*. P3 C

Other observations included whether patients came in with a neck collar:

*… that’s the other thing, if they come in in a collar, then that would definitely increase the suspicion. Sometimes you go through the history and you think about it and if they’re in a collar, I think that kind of makes you more likely to go with a CT scan rather than not. I guess there’s lots of clues along the way*. P4 R

*So I guess they come in with a C-Spine collar … you could argue is that part of the pre-test probability? But you’ve already got that cognitive bias, we need to get a C-Scan perhaps. But, in my mind, we’ve still got to do a clinical assessment from scratch and still assess these patients ourselves.* P17 C

Handover information from paramedics was another important point of reference in gaining an understanding of a situation:

*patients that have a significant mechanism that come in collared already at the scene, because of either some tenderness or a significant mechanism that the QAS [Queensland Ambulance Service] Officers felt warranted some mobilisation would always make me a bit more hyper alert because somebody at the scene has seen them assessed and thought this needs to be applied. … I think I would rely heavily on the QAS handover in terms of what they witnessed at the scene. Whether they managed to speak to anybody, if anybody or his friends or her friends knew what had happened*. P1 R

Other collateral information included contacting nursing homes or family for details about the incident and patient background:

*You could get some collateral from the nursing home, have a chat with the nurses looking after her and just say, ‘look, how has she been? Have you noticed any changes in her behaviour, in her personality, her pain scores’ and that kind of stuff*. P5 R

The impact of assault or potential domestic violence on the decision-making process was considered by participants as the patient might be downplaying or overstating the injury and hence a scan might provide more objective information in that situation:

*cases where someone’s been involved in domestic violence and those kinds of things, I would always be more careful in my decision-making with imaging. … if you’ve got somebody that’s been a victim of domestic violence, there’s lot of factors which might influence the way they tell you the story or what they tell you. … normally we would rely heavily on the history, but sometimes that might not be freely available and if you’ve got objective evidence of somebody with lots of external bruising then I would be more inclined to scan, just in case – it would depend on the individual case – but in case I wasn’t getting the full story. Then I wouldn’t want to miss anything that could potentially become life-threatening or disabling*. P1 R

CT scans were perceived by some as more objective and reliable than other sources of information:

*but I suppose sometimes if there’s any unreliable part of a story or your examination, then a scan is good objective evidence. If somebody has no neurology but some symptoms, then it’s about trying to minimise risk for the patient and yourself*. P1 R

*If there’s any doubt of any of those things, I would be more erring on the side of getting a scan.* P2 R

*I think it's because patients with a headache frequently come up in morbidity and mortality meetings, as in people with strange diagnoses that are missed, and then people always feel like, why weren’t they scanned when they had their initial symptoms?* P7 C

### Holistic patient-centred care

The importance of patient-centred and holistic care and involving patients in decisions was evident:

*You give your best recommendation to the patient and they have to decide whether they want the scan or not. If it's a child, obviously, it’s the parents*. P12 R

*There's a little bit then of clinical decision making and I often share that decision making with patients and their relatives as to the risk benefits*. P13 R

Participants highlighted the need to involve patients in decisions in two particular situations: firstly, to inform about radiation risk when the clinical indication for CT is not clear; and secondly, when patients were very anxious:

*the patient said, look I understand everything, I feel fine, I'm with my husband, I'm with my son, you've explained everything clearly and if there's a problem I can come back, but I would rather not have that. I thought that was a sensible decision-making process. But I think rather than being something completely paternalistic, I'm happy to give a guidance or recommendation that if a patient felt very strongly one way, I wouldn't necessarily dig my heels in.* P13 R

*If she’s someone who’s really, really worried about this and is going to keep coming back until such a time as someone images her neck, then there might be a role for shared decision-making, where you say, look, this is the risk of radiation to your thyroid and et cetera*. P17 C

Other sub-themes emerging under holistic patient-centred care are summarised in Table 4:

- *Medical seeking behaviour*
- *Baseline level of risk*
- *Ability to look after themselves*
- *Patient preferences*
- *Consultations with family*
- *Proximity from medical services*

**Table 4:** Sub-themes relating to shared decision-making and holistic patient-centred care

Medical seeking behaviour	*I mean social factors I would take in as well. I've worked in a country hospital and you have pretty low thresholds to scan certain people because they don't really seek medical care all the time. If you know that they come in it's probably something bad and you shouldn't just go by a single algorithm which has been run by a different [city] population with that population.* P21 R
Baseline level of risk	*The decision making should be not about whether they're on it [antiplatelet], but whether you think there's a risk of them having a bleed and whether you would act on that risk. … But now irrespective whether it's aspirin, [anti]platelets or [anticoagulant], it's not about that, it's about judging their risk. So that's changed- whereas previously I did have to read that line and my risk stratification was a lot higher*. P3 C *Frailty is independent of age. So, you can have an osteoporotic 60-year-old female which I'd scan, but you could have a fairly robust 70-year-old male that you would have a lower threshold of scanning. I think taking into account how likely you think a fracture in the individualised patient is important too*. P7 C
Ability to look after themselves	*So I think it's kind of tying all of those factors in together at the same time. But then also I think some of the other things that sometimes make a difference is also how safe that patient themselves is. So for example, in certain cases if they live very close to the hospital, they have a robust support system, they're capable of making sensible decisions and we know that they will be able to seek medical attention appropriately, then that sometimes gives more of a leeway of monitoring the patient rather than going straight for a scan. Especially when they're sitting on the cusp. But if we don't have those resources or they want to leave the hospital or they're socially vulnerable, there's nobody actually able to look after them to flag if they're suddenly behaving differently and need medical attention, that might lower my threshold*. P3 C
Patient preferences	*You do sometimes over-investigate on the basis of patient preference. In those cases you always discuss with the individual why we wouldn't scan. You tell them that there's a small but real risk that they may end up with cancer as a result of radiation injury. There are some patients that are just fixated on, whether or not it's through their own anxiety or their own issues, they're fixated on getting a test. While we're not obligated to do it, at times it's just for everyone's benefit, you just do it*. P5 R *sometimes in very, very difficult situations, we end up doing the scans, just for appeasing the patients. It does happen. It does happen. Because sometimes the patients are difficult and they don’t want to go and they're like ‘can I have this, I can't go to my GP’*. P8 C
Consultations with family	*So that would the point that I would be discussing with the family, going I don't really think this is going to be helpful, apart from delaying her transit through ED, which way would you like. I think that's completely a shared decision-making with the family and the patient*. P3 C *I do that in consultation with family. … I'll call the next of kin if they're not there, I'll explain to them that he might have a bleed on her brain, she is on aspirin, there are no signs of it at present and there's no reason to do a CT scan because no-one would operate on her because of her age, however if they strongly want it done, I usually do it and that's pretty much to avoid complaints and medical legal problems. But I've never had that happen. Usually I explain it to them and they say, ‘oh that's okay, we'll take nana home’ and they know if her GCS [Glascow Coma Score] drops in the next couple of days, it'll be because she's had a bleed in the brain and that that's not a bad way to go and then they go home*. P6 R
Proximity from medical services	*Then you also take into account patient disposition, how far away do they live from the hospital, do they have someone to look over them? …. Going back to if they do live a long way from the hospital you probably have a lower threshold to scan them given that they might not be able to present quickly in case of any change in their mental status. Then also whether or not they've got someone to keep an eye on them, just to pick up if they start acting a little bit confused and all that kind of stuff*. P5 R

### COM-B analysis

Mapping of the sub-themes against the COM-B framework showed that the behaviour change components of the COM-B model were integrated and played a role in decision-making (Table 5). Although the focus of the study was not on behaviour change, the analysis revealed participants’ capabilities, opportunities and motivations relating to CT ordering and how these could inform future interventions.

**Table 5:** Mapping of sub-themes against the COM-B System [37]

COM-B				Sub-theme
**Behaviour System**	**Sources of behaviour**	**Intervention functions**	**Policy categories**	
**Capability**	Psychological	Modelling Environmental restructuring Restrictions Education Persuasion Incentivisation Coercion Training Enablement	Fiscal measures Guidelines Environmental/ Social planning Communication/ Marketing Legislation Service provision Regulation	Improved awareness of cost considerations Consult with senior doctors Consult with radiology staff Clinical decision support tools Personal experience Professional judgement Professional responsibility Clinician gestalt Model consultants and more experienced doctors Ongoing training National health system Resource considerations Bed flow considerations Patient safety Patient management Medico-legal issues Holistic patient-centred care
	Physical			
**Opportunity**	Social			
	Physical			
**Motivation**	Reflective			
	Automatic			

## Discussion

This qualitative study explored the influences on ED doctors’ ordering of CT head and C-spine. Thematic analysis of the data showed five main themes namely 1) health system and local context, 2) work structure and support, 3) professional practices and responsibility, 4) reliable patient information, and 5) holistic patient-centred care. The identified themes and sub-themes highlight that CT ordering is complex and multifaceted; hence change requires a continuing effort within a supportive structure. The mapping of sub-themes against the COM-B model provided a useful basis to develop potential future interventions most likely to achieve behavioural change.

The findings of this study suggest that the decision to order a CT is influenced by numerous factors from the health service/setting, interactions that occur within the health setting down to factors related to the individual doctor and their patient. Differences between health systems and availability of local resources may have played a role in the ordering of scans. Our participants all worked in a public hospital in Australia, where ED assessment and management (including scans) is completely covered by the national insurance scheme. These possible differences in ordering practices between settings suggest a need for improved understanding amongst clinicians to rationalise limited resources considering health budgets are capped. Participants relied on support structures in place to guide decisions with junior doctors relying on senior doctors for support and acting as role models. Whilst participants considered patient safety aspects the use of clinical decision support tools and the overall integration of these to guide decision-making varied. As expected, doctors indicated that professional responsibilities and judgement were ingrained in their clinical decisions, facilitating patient-centred care.

The results showed varied approaches to the use of clinical decision support tools with some doctors using it as a checkpoint following a decision already made to order or not order a scan. This is similar to a Canadian study that explored ED doctors’ diagnostic processes and reasoning processes that found that doctors applied clinical decision support tools after they had already decided to order a test [40]. This highlights a need to evaluate the barriers and facilitators affecting the use of clinical decision support tools and how these tools could be integrated into workflow to support clinicians’ decision making [41]. The results also showed inconsistencies in which decision aids were used in specific scenarios. These findings show a need for ongoing education in the application and workflow integration of clinical decision support tools in the hospital context, e.g., using through vignettes [42].

CT scans were ordered in cases when doctors were unsure about the seriousness of an injury, specifically when collateral information was not available or considered to be unreliable. In situations of diagnostic uncertainty participants reported ordering a CT scan to provide objective information to guide management decisions. This practice might lead to overuse of CT scans. Considering that no decision support tool is 100% sensitive and specific, there is a need to determine an acceptable threshold for imaging which does not significantly reduce the diagnostic yield. This can be addressed through educational programs incorporating evidence showing decreased ordering did not result in increased death or missed diagnosis [43] and integration of pre-test probability (the estimate of the probability of a patient having a disease prior to testing) [44]. Other successful strategies to increase doctors’ awareness of factors that impact on their use of imaging included addressing cognitive bias such as knowledge gaps, risk aversion, confirmation bias as well as addressing poor coordination between specialties and commercial pressures [45]. Interventions to address cognitive biases linked to low value interventions include audit and feedback, while shared decision-making is one strategy that can be used by clinicians to managing clinical uncertainty [46, 47].

Although consultants indicated their practices were not driven by fear of litigation, the impact of patient and carer expectations were emphasised as important considerations. This finding is similar to an American focus group study with ED doctors and patients on the ordering of CT scans and cognitive task analysis that identified patient expectations, establishing trust, anxiety (patient and provider), constraints related to ED practice and the influence of others played a role [48]. A recent survey of specialists, hospital staff and general practitioners (GPs) pointed out patient expectations, potential for medical litigation and uncertainty of diagnosis as reasons for requesting unnecessary medical tests [49]. Barriers to reduce head and C-spine CT orders identified in a Canadian study that involved semi-structured interviews with ED doctors were linked to beliefs about consequences; beliefs about capabilities; behavioural regulation; memory, attention and decision processes; environmental context and resources; and social influences [50].

Our study showed that many issues are considered by ED doctors in deciding when to order CT head and c-spine scans with patient safety at the centre. Although most participants considered the potential radiation harm caused by unnecessary testing only few considered the impact on resources (financial, equipment and manpower) and incidental findings. This finding suggests a need for better awareness amongst ED doctors of the potential impact of unnecessary scans on patients and the health system: an awareness of the impact of low value care and that health funding is not unlimited. A recent systematic review on interventions aimed at reducing low-value health services found that multicomponent interventions addressing both patient and clinician roles in overuse had the greatest potential to reduce low value care with strategies such as clinical decision support, performance feedback and provider education with solid evidence when paired with other strategies [51]. The results from this study will inform ongoing local, regional and national strategies to address the ordering of CT scans to avoid provision of low value care.

### Strengths and limitations

The research team consisted of clinicians with complementary skills and experience which facilitated meaningful discussions and interpretation of the data: clinical research and evidence-based practice, senior and junior ED doctors, senior radiologist, senior neurologist and a nurse in senior management. Although the interview period had to be extended over an 11-month period due to the COVID-19 pandemic to allow interviewing enough ED doctors to reach saturation, this did not impact on the outcomes. Face-to-face interviews helped to foster a relationship between the interviewer and interviewee, enhancing the depth of information obtained. The methodological approach was structured and transparent with all participants provided an opportunity to review their verbatim interview transcripts.

The research focused on the perceptions of ED doctors in two Australian public hospitals and the results may not be translatable to other ED doctors. Also, the findings may not apply to other settings with different models of care are in place. Our study was conducted following the introduction of an electronic medical record system however the impact of this change was not explored in the interviews as the focus was on current practices.

## Conclusion

The interviews highlighted that multiple factors are considered by ED doctors during decisions to order CT scans for head or c-spine injuries. Results identified a need for increased education on the use of clinical decision support tools to achieve more consistent use of the tools between clinicians. Additionally, as imaging requests are increasing locally, nationally and internationally, strategies to improve awareness on low value care to address overestimating of the benefits and underestimating the harm of CT scans are needed as well as processes to better triage the highest priority patients. Interventions aimed at reducing the ordering of scans will need to be multifaceted in order to embed changed behaviour into business as usual and incorporate clinician capabilities and motivation as well as local initiatives and the policy framework. The insights obtained through this study will be used to co-design, develop and trial interventions to optimise appropriate and evidence-based ordering of CT scans for head injuries.

## Supplementary information

**Additional file 1:** COREQ (COnsolidated criteria for REporting Qualitative research) Checklist.

**Additional file 2:** Evaluation of the ordering of CT scans of head and cervical spine Semi-structured Interview Questions.

## Data Availability

De-identified datasets used and/or analysed during the current study are available from the corresponding author on reasonable request.
