# Supplementary material for: Ordering of computed tomography scans for head and cervical spine: a qualitative study exploring influences on doctors’ decision-making
Source: BMC Health Serv Res. 2022 Jun 18;22:790. doi: 10.1186/s12913-022-08156-2 (PMC9206095; doi:10.1186/s12913-022-08156-2)
Supplement: Supplementary file 2 — Additional file 2: Evaluation of the ordering of CT scans of head and cervical spine Semi-structured Interview Questions. [file 12913_2022_8156_MOESM2_ESM.pdf]

Evaluation of the ordering of CT scans of head and cervical spine

Semi-structured Interview Questions

Opening Remarks:

"Thank you for your time.

I would like to start off with a brief introduction of myself: My name is ___________________________ and I am [describe role]

The purpose of this interview is to explore aspects that influence doctors' practices and decision-making in the ordering of CT scans. This will help us in better understanding the ordering of scans and barriers and facilitators in following clinical decision tools to inform the development and implementation of sustainable strategies.

We really value your input and honest contribution. Anything you tell us will be treated with strictest confidence and your identity will be protected.

If you have any questions regarding this research please do not hesitate to contact any of the research team members.

The interview should take about 30 minutes.

Pre-interview information to be collected:

1. Gender: ☐ Male ☐ Female ☐ Other
2. Age group (years): ☐ 21-30 ☐ 31-40 ☐ 41-50 ☐ 51-60 ☐ ≥ 61
3. Country/region of birth: ☐ Australia ☐ China ☐ Malaysia ☐ New Zealand ☐ United Kingdom ☐ Other, please specify ________________
4. At which university did you obtain your initial medical qualification? ____
5. Which year did you graduate with your medical degree? ___________________
6. In which year did you first obtain your registration to practise as a doctor in Australia? ______________
7. How many years have you worked as a doctor? ___________________
8. How long have you worked as an ED doctor? (months or years) ___________________
9. If you have any specialist training, please list these ____
10. When did you obtain your specialist training? ___________________

Before we begin, I would like to learn more about you.

1. Can you please provide a brief outline of your current position at Gold Coast Health and what this involves?

    - How long have you worked in this position?
    - What are your current roles? If you have multiple roles, please specify these roles e.g. training of junior doctors, research, etc.
    - What proportion of your time is spent on your various roles?

The next few questions will focus on your decision-making process when ordering of CT scans for adults, therefore patients who are 16 years and older.
Note that we are referring to non-contrast CT scans for trauma-related reasons (therefore not spontaneous headache and altered level of consciousness or suspicion of stroke).

2. Can you please describe for me the decision-making process that you go through to order:

    a. A CT scan of the head

    b. A CT scan (not angiogram) of the cervical spine (C-spine)

3. Considering your ordering of CT scans of head or C-spine:

    - What role do various clinical decision tools play in your decision-making process? How do you use the tools?
        - Which tools do you consider?

    - What else do you consider during your decision-making process?
        - Patient factors (e.g. age ….)
        - Diagnosis

    - Other than the patient, what influences your decision-making process?
      Prompts:
        - Cost
        - Departmental pressures/factors such as bed block/NEAT targets
        - Supervisor factors
        - Medicolegal concerns/risk avoidance
        - Political factors

4. **Consider the following scenarios:**

a. A 19-year-old was found unconscious at a party. He has normal vital signs, no sign of trauma and no clear neurological deficit. There is no witnessed drug intake, but he smells of alcohol.

- What are your considerations for imaging his brain (head).
- Would you proceed to ordering a scan?

b. A 43-year-old driver of a car was rear-ended at 40km/h. She was wearing a seatbelt and she could extricate herself from the car and walked around on the scene. She never lost consciousness, has no neurology but now complains on midline C-spine tenderness. She declines analgesia and says the pain is 4/10.

- What are your considerations for imaging the C-spine?
- Would you proceed to ordering a scan?

- How will your decision be impacted if she presents to the ED two days after the car accident?

c. An 87-year-old nursing home patient has had an unwitnessed fall and is found on the floor. There are no clear injuries. The patient has a background of dementia and her cognitive function seems unchanged. She is on aspirin.

- What are your considerations for imaging her brain?
- Would you proceed to ordering a scan?

- What would you do if she presents one day after the fall?

- How will your decision be impacted if she represents after 7 days due to ongoing headaches?

5. Consider a recent ordering of a CT scan for the head or C-spine when you were unsure whether a scan was necessary.

What process did you go through?

5. Can you describe for me whether you have recently made any changes to your CT scan ordering decision-making processes?

   Prompts:
   - Something that you used to do that you no longer do, something that used to be your standard practice
   - Please describe the impact of the change(s). How important do you consider the change(s) to be?
   - What initiated this change?
   - What did you consider when deciding to change your practice?
   - What were the facilitators that you encountered during this process?
   - What frustrations/barriers did you encounter during this process?
   - How did you feel about making this change?

The next few questions will focus on the ordering of CT scans in general.

6. Can you tell me about scenarios/situations that involve particular patient groups who are referred for scans due to doctors having a suspicion of a condition but who may benefit from more rational test ordering.

   Prompts:
   - TIA stroke
   - Minor head injury
   - Headache without neurology
   - Chronic abdominal pain
   - Whiplash neck pain
   - Kidney stone

With reference to what we have already discussed, we will now talk about aspects that might assist or facilitate decision-making processes.

7. What are your thoughts about clinical decision tools being incorporated into ieMR?
   Prompts:
   - How should this be implemented?
   - Can you give examples?
   - What about clinician pop-up fatigue?

8. What are your thoughts about the relationship between clinical decision tools and clinical judgement?
   Prompts:
   - What are appropriate reasons to deviate from clinical decision tools?

10. Do you have any further comments about decision-making in the ordering of CT scans that we have not covered?

Thank you for your time.
